# Influence of silicon nano-particles on *Avena sativa* L. to alleviate the biotic stress of *Rhizoctonia solani*

**DOI:** 10.1038/s41598-023-41699-w

**Published:** 2023-09-14

**Authors:** Faiza Ahmad, Khajista Jabeen, Sumera Iqbal, Aisha Umar, Fuad Ameen, Marek Gancarz, Doaa Bahaa Eldin Darwish

**Affiliations:** 1 Department of Botany, Lahore College for Women University, Lahore, Pakistan; 2 Institute of Botany, University of the Punjab, Lahore, Pakistan; 3 Department of Botany and Microbiology, College of Science, King Saud University, Riyadh, 11451 Saudi Arabia; 4 Faculty of Production and Power Engineering, University of Agriculture in Krakow, Balicka 116B, 30 149 Krakow, Poland; 5 Institute of Agrophysics, Polish Academy of Sciences, Doświadczalna 4, 20-290 Lublin, Poland; 6 Botany Department, Faculty of Science, Mansoura University, Mansoura, 35511 Egypt

**Keywords:** Microbiology, Plant sciences, Biogeochemistry

## Abstract

*Avena sativa* L. a cereal crop that is badly affected by several abiotic and biotic stresses. In the current study, silicon nanoparticles are used to mitigate the harmful effects of root rot disease caused by *Rhizoctonia solani* Kuhn on the growth of *A. sativa*. In vitro (Petri plates) and in vivo (pots experiment) were performed to measure the various physiological and biochemical parameters i.e. osmotic potential, chlorophyll, proline content, growth parameters, sugar, fresh and dry weight, and disease index. Results revealed that physiological and biochemical parameters were reduced under fungal stress with silicon nanoparticles treatment as compared to the control group. Si nanoparticles helped to alleviate the negative effects caused by fungus i.e. germination percentage upto 80%, germination rate 4 n/d, radical and plumule length was 4.02 and 5.46, dry weight 0.08 g, and relative water content was (50.3%) increased. Fungus + Si treatment showed the maximum protein content, i.e. 1.2 µg/g as compared to Fungus (0.3 µg/g) treated group. The DI was maximum (78.82%) when the fungus directly attacked the target plant and DI reduced (44.2%) when the fungus was treated with Si nanoparticles. Thus, silicon nanoparticles were potentially effective against the stress of *R. solani* and also used to analyze the plant resistance against fungal diseases. These particles can use as silicon fertilizers, but further studies on their efficacy under field conditions and improvement in their synthesis are still needed.

## Introduction

*Avena sativa* L. commonly known as, ‘Oat’ is a member of the family Poaceae. It is herbaceous, annual, self-pollinated grass of Rabi season. Among the other plant cereal species i.e. *Triticum* sp*. *(wheat)*,* barley *(Hordeum vulgare), Oryza sativa* (rice), maize (*Zea mays*), and *Sorghum bicolor *(sorghum) it ranks sixth in the world cereal production statistics^1,2^. Oats are annual grasses and are the most important cereal for human and animal consumption. *A. sativa* is full of nutritious cereal containing soluble fiber in high concentration, especially beta-glucan, vitamins, antioxidants, and minerals^3,4^. Oats contain avenanthramides, which are unique antioxidants therapeutically effective in heart-related diseases and in lowering blood sugar and cholesterol level due to the presence of beta-glucan^5^. In livestock oats consumption is highest approximately 74% of the total world’s usage^6^.

Worldwide agricultural productivity is subjected to increase environmental constraints in the form of abiotic and biotic stresses that badly influence plants^7^. Oats fulfill the double requirement as compared to other fodders^8^. Many abiotic and biotic factors are responsible for minimum production in the world. Diseases attacking crops are also the major reason for production loss^9^. Oats are susceptible to many diseases like crown rust, stem rust, leaf spot, leaf blotch, root rot, stem rot, etc. Several phytopathogens (*Puccinia coronate*, *Puccinia graminis, Pyrenophora chaetomoide, Rhizoctonia soloni, Fusarium graminearum*) are highly damaging at any stage of *Avena sativa* crop. The biotic stress due to fungal pathogens is a major cause of yield loss in subtropical and tropical regions^10^.

*Rhizoctonia solani *Kuhn., a phytopathogen belongs to Ascomycetes. This fungus was explored 100 years ago by Kuhn. This is a soil-borne pathogen with a threadlike growth pattern. The wide host range (wheat, barley sorghum, oats, etc.) is the specialty of this fungus. The pathogenic action causes various harmful diseases in plants i.e. damping off, collar rot, wire stem, and root rot. The early developmental stage like seedling is more susceptible to this harmful fungus and causes severe plant loss by attack on lower stems and roots of plants^11,12^. *Rhizoctonia* root rot is a common oat fungal disease caused by *R. solani* and 10–15% yield reductions in dryland cereal productions^13^.

Fungal diseases are controlled by traditional and conventional fungicides as well as introducing resistant varieties, which reduce the severity of diseases^14,15^. The resistance is controlled by the genetic diversity of fungal pathogens as well as by genotype with environment interaction^16^. Excessive use of fungicides causes significant economic loss, pathogens resistance, and many harmful environmental effects^17^. There is a need for more effective alternative solutions for fungal pathogen management. Nanotechnology is an emerging field, in which nano-sized materials are used for plant growth^18^. These emerging critical issues poses the threats to food security. While the food demands in the world has increased steadily and become more severe as world population increase^19^. Current advancement in the usage of NPs, which can cause substantial increase in ‘seed germination, rate of photosynthesis and antioxidant enzyme activities’^20^. Silicon NPs gained the special attention due to their quasi-essential effects on plants and playing an important role in alleviating the environmental stresses^21^. Nanoparticles offer environmentally safer substitutes for chemical fertilizers without endangering the environment. SiNPs offer practical answers to a variety of agricultural issues regarding weeds, pathogenicity, pest infestation, abiotic stresses, loss in crop production with targeted delivery^22^. Nano Si are more effective in reducing heavy metal toxicity, UV-B stress and providing more yield^23^.

Silicon nanoparticles (Si NPs) range from 1 to 100 nm in size^24^. This influences the stomatal movement and transports DNA (other chemicals) to isolate the plant cells and intact leaves. Si-NPs have a beneficial impact on the agriculture sector and they may improve yields, leading to increase productivity^25^. Silicon nanoparticles also mitigate the (biotic) stress in monocot plants^26,27^. Technologies and tools for investigation and transformation offer by nanotechnology develop the biological systems to mitigate the biotic and abiotic stresses in organisms^28^. Nanotechnology is the understanding and control of matters, where unique phenomena enable the plants to build a mechanistic approach on behalf of the novel applications of the nanoparticles in stressed environment for plants survival^29^. Applications of nanotechnology activate the number of defense mechanisms in response to stresses in plants, which enhance the tolerance against the adverse conditions^30,31^. The prospective uses of nanoparticles in the agricultural sector, namely the growth and reduction of a (biotic) stress tolerance in plants to achieve the sustainable agriculture, by using distinctive features of various nanoparticles and their influence on crop plants^32^. Interactions between environmental factors (biotic and abiotic) and the internal mechanisms of plants created the tolerance by the shielded effects of nanoparticles to grow successfully^33^. Nano technique improve the morphological and nutrient profile of the plants under stresses by triggering the ‘physiological and genetic’ repair mechanisms^34^. This technology develops the resistance in plants to combat the stresses^35^.

Worldwide agricultural productivity is subjected to increase environmental constraints in the form of abiotic and biotic stresses that badly influence plants. The biotic stress due to fungal pathogens is a major cause of yield loss of *A. sativa*. Pathogenic action causes various harmful diseases in plants. The early developmental stage like seedling is more susceptible to this harmful fungus and causes severe plant loss. Silicon nanoparticles (Si NPs) have a beneficial impact on the agriculture sector and also improved the growth of our plant yields, leading to an increase the productivity.

This study aimed to evaluate the effects of silica nano-particles in the mitigation of biotic stress caused by *Rhizoctonia solani* in *Avena sativa* (oats). These particles also mitigate the biotic stress of *R. soalni* in our test plant species. The morpho-physiological attributes were also studied by Pearson’s Correlation and pathogenic fungi identified in ITS based phylogenetic study.

## Materials and methods

### Source of target plant

Current experiment was performed in the Fungal Biotechnology and Plant Physiology Lab, Department of Botany, LCWU, Lahore (Latitude: 31°32 N; Altitude: 688 ft above sea level), Pakistan during oats growing season (December 2017-March 2018). Seeds variety (NARC-Oats) of oat were obtained from NARC (National Agriculture Research Center).

### Culturing and molecular identification of Rhizoctonia solani

The *Rhizoctonia solani *cultures were prepared by isolation from diseased potato tubers by a specific culturing method. For this purpose, sclerotia were scratched from potato tuber and dipped in spirit for 10–15 sec. The removed sclerotia were soaked in d.H_2_O for 10–15 sec and placed on sterilized filter paper. Moister was absorbed from sclerotia into filter paper and then sclerotia were transferred to Potato Dextrose Agar (PDA, Merck) and placed in an incubator at 23 °C. After 2 days’ mycelial threads were isolated and placed on new PDA media for sub-culturing. The culture was identified by DNA sequencing of ITS regions. The modified CTAB procedure was used to extract the total genomic DNA of the specimen^36^.

The nuclear ribosomal ITS region was amplified using the primers ITS1 (5′ CTTGGTCATTTAGAGGAAGTAA′3) and ITS4 (5′TCCTCCGCTTATTGATATGC′3)^37^. The amplification conditions were 35 cycles of 95 °C for 30 s, 52 °C for 30 s, and 72 °C for 1 min, followed by a final extension at 72 °C for 10 min. The consensus sequences were generated in BioEdit version 7.2.5^38^ and homology searches were performed at the National Center for Biotechnology Information (NCBI) using BLASTn. The generated sequences were deposited in GenBank and accession numbers were assigned. For identification and confirmation of this fungal specie, a phylogenetic tree was constructed.

### Synthesis of ‘Si’ nanoparticles

Zulfiqar et al*.*^39^ method was used to synthesize the silicon NPs from sodium silicate under alkaline conditions. This method is simple in synthesis, thermally stabile, great biocompatibility, and low in toxicity. A mixture containing ethanol and ammonia (E/A) in equal amounts (30 mL each) was prepared. The 0.5 mL Sodium Silicate Solution (SSS) was added in d.H_2_O (7 mL). The SSS solution was added drop-wise to the E/A mixture and allowed for 1 h. After aging this, centrifuged the SSS+E/A solution and washed by using d.H_2_O. The resulting silica nanoparticles were dried. This process is a streamlined method, safer, simpler and more cost-effective. The aqueous suspension of SiNPs analyzed using wavelength scan from 200 to 800 nm in UV–Vis spectrophotometer UV analysis characterized and confirmed their particles presence.

### In vitro-plate method

The seeds of the oat were surface sterilized by using sodium hypochlorite (5%) for ten minutes and then rinsed with d.H_2_O (4 times). Petri plates were sterilized in an electric oven at 170 °C for 1 h. The ten seeds were placed randomly in sterilized Petri plated and moistened by using respective treatments. The randomized complete block design (RCBD) was used in this study. Treatments were divided into control (without any treatment), fungus, silicon, and Fungus+Si with three replicates. In the next stage fresh culture of *R. solani* + 1.5 mM nano-silica was used and compared with control (d.H_2_O). Seed germination was observed for six to seven days. Data for the germination rate of each treatment was noted after 24 h. After seven days’ root length (cm), and shoot length (cm) were noted from each of the replicates. Readings were taken after seven days of seedling growth in Petri plates. A few *in vitro* parameters like “Germination percentage, Germination rate^40^, Seed vigor of seedlings^41^, and Tolerance index of seedlings^42^ through plate method were calculated by following formulas:

1 $$ {\text{Germination }}\% {\text{ age }} = \frac{{{\text{No}}.{\text{ of seeds germinated during the time interval}}}}{{{\text{Total no}}.{\text{ of seeds sown}}}} \times 100, $$

2 $$ {\text{Germination rate }} = \, \Sigma {\text{g}}/{\text{t,}} $$

g = Percentage of seed germination every day, t = Total germination period.

3 $$ {\text{Seed Vigor }} = {\text{ seedling length }} \times {\text{ germination percentage}}, $$

4 $$ {\text{Tolerance Index }} = \frac{{\text{Mean root length in the treatment}}}{{\text{Mean root length in the control}}} \times 100. $$

### In vivo-pot experiments and soil characteristics

This part of the experiment was performed for the physiochemical analysis. The experiment conducted in pots (30 cm) was organized in Randomized Complete Block Design (RBCD) and comprised five sets of treatments (with three replicates). Each pot was filled with 10 kg of soil and twenty seeds. Set A was comprised of sterilized soil and B of unsterilized soil. Set C comprised sterilized soil and fungal inoculum, while D with irrigated soil and silicon nanoparticles solution. Set E consisted of fungus suspension and silicon nanoparticles solution. Set A and Set B was considered positive control (without any treatment) and negative control (Commercial fungicide; Thiophanate methyl), respectively. For control sets (A and B), soaked seeds in d.H_2_O were sown in the pots. The seeds of C and E were surface sterilized by using a 5% sodium hypochlorite solution and then washed with d.H_2_O. These seeds (Set E) were coated with fungal spore suspension (1 × 10^5^ conidia per mL) of *R. solani* for 3 h. The 5 mM nano silica solution was added to each pot of D and E. Sampling was done at the reproductive stage after 85 days of germination of seeds. All samples were weighed carefully and saved in the refrigerator for physiochemical analysis.

Soil texture was determined accordingly Brady and Weil^43^. Soil texture (amount of sand, silt, and clay) was determined by using a textural triangle.

$$ {\text{Percentage of sand}},{\text{ silt}},{\text{ and clay }} = \,\frac{{{\text{CHR}}}}{{{\text{Wt}}.{\text{ of soil}}}} \times 100, $$

where CHR is “Corrected hydrometer reading”.

For physiological characteristics, the soil sample was air dried, and then 5 g of it was stirred with 50 mL d.H_2_O to form a uniform mixture. The EC and pH of this solution were recorded with an EC meter and pH meter respectively. The following physiochemical parameters were performed and estimated at the reproductive stage of the plant including “Relative water content, Osmotic potential, Membrane stability index, Disease index, Chlorophyll and Carotenoid contents, Sugar content, Proline content, and Protein content”.

### Biochemical and physiological parameters

#### Relative water content

Relative water content was determined by following the method of Wheatherley^44^ using the following equation:

$$ {\text{RWC}}\% = \frac{{{\text{Fresh weight }}{-}{\text{ Dry weight}}}}{{{\text{Turgid weight}} - {\text{ Dry weight}}}} \times 100. $$

#### Osmotic potential

The osmotic potential was measured accordingly Capell and Dörffling^45^. For this purpose, the leaf was enclosed in a disposable syringe (5 mL) and kept in the refrigerator. Sap from the leaf was squeezed out from defrosted leaf, and then osmotic potential was calculated by the following formula:

$$ {\text{Osmotic potential }} = \, - {\text{ osmolality }}\left( {{\text{mosmol}}} \right) \, \times \, 0.{831} \times { 1}0 - {5 } \times {\text{ T }}\left( {\text{K}} \right). $$

#### Membrane stability index (MSI)

Membrane stability index was measured accordingly modified method of Sairam^46^. The 0.1 g leaf was weighed, washed, and dipped in d.H_2_O (10 mL) in a test tube. All samples were placed in a water bath for 30 min at 40 °C and then EC (electrical conductivity) was measured. After that all samples were again placed in a water bath at 100 °C and EC was noted again.

$$ {\text{MSI }} = \, \left[ {{1 }{-} \, \left( {{\text{C1}}/{\text{C2}}} \right)} \right] \, \times { 1}00. $$

#### Disease index

For measurement of the disease index, three plants were taken from each pot and the fresh weight of roots and root length were recorded^47^. Sampling was done at the reproductive stage approximately after 80–90 days and different yield parameters were measured i.e*.* number of branches, plant height, and weight, spikelet’s number, and seeds number. For the determination of the disease index following formula was used.

$$ {\text{Disease index }} = \, \left\{ {{1}00 - \, \left( {{\text{weight of diseased roots}}/{\text{ weight of roots}}} \right)} \right\} \, \times {1}00. $$

#### Chlorophyll and carotenoid contents

Chlorophyll contents were extracted accordingly Hiscox, and Israelstam^48^ method. Absorbance was measured by using a spectrophotometer of both blank and extract at 470, 645, and 663. The chlorophyll content was calculated by formulas of Arnon^49^, Lichtenthaler and Wellburn^50^ followed to estimate carotenoid content.

$$ {\text{Chlorophyll a}} = \, 12.21 \, \left( {{\text{A}}663} \right) \, - 2.81\left( {{\text{A}}645} \right), $$

$$ {\text{Chlorophyll b }} = \, 20.13 \, \left( {{\text{A}}645} \right) \, - 5.03\left( {{\text{A}}663} \right), $$

$$ {\text{Total chlorophyll }} = \, 20.2 \, \left( {{\text{A}}645} \right) \, + \, 8.02 \, \left( {{\text{A}}663} \right) $$

$$ {\text{Carotenoid content}} = \frac{{\left[ {1000 \, \left( {{\text{A}}470} \right) \, - \, 3.27\left( {\text{chlorophyll a}} \right) \, - \, 104\left( {\text{chlorophyll b}} \right)} \right]}}{227}. $$

#### Sugar content

Sugar content was determined by using Dubois^51^ method. The plant material (0.5 g) was ground after adding 10 mL d.H_2_O and centrifuged for 5 min at 3000 rpm. In 0.1 mL supernatant, 1 mL phenol (5%) was mixed thoroughly. This mixture was incubated for 1 h at room temperature and then conc. sulphuric acid (5 mL) was added. The absorbance was checked at 420 nm. A standard curve for glucose was used for the estimation of sugar content.

#### Proline content

The proline content of plant samples was estimated using upper leaves (2nd and 3rd) and Bates et al.^52^ method was followed. Sample proline content was checked with the help of the proline standard curve.

#### Protein content

Lowery et al.^53^ method was followed for protein content. The protein content was noted regarding the standard curve.

### Statistical analysis

In this study, the data were analyzed by using Statistix 8.1 under factorial design with three replications following Steel et al.^54^. The mean values of data were tested with the least significance difference (LSD) test at the probability level of 5% (P ≤ 0.05).

The correlations between the variables under investigation were measured using Pearson's correlation analysis. Using Rstudio software, coefficients for principal component analysis (PCA), and Pearson correlation analysis was also calculated between the observed data for *A. sativa*.

### Statement

All the methods were carried out in accordance to international guidelines and regulations.

## Results and discussion

**Figure 1:**
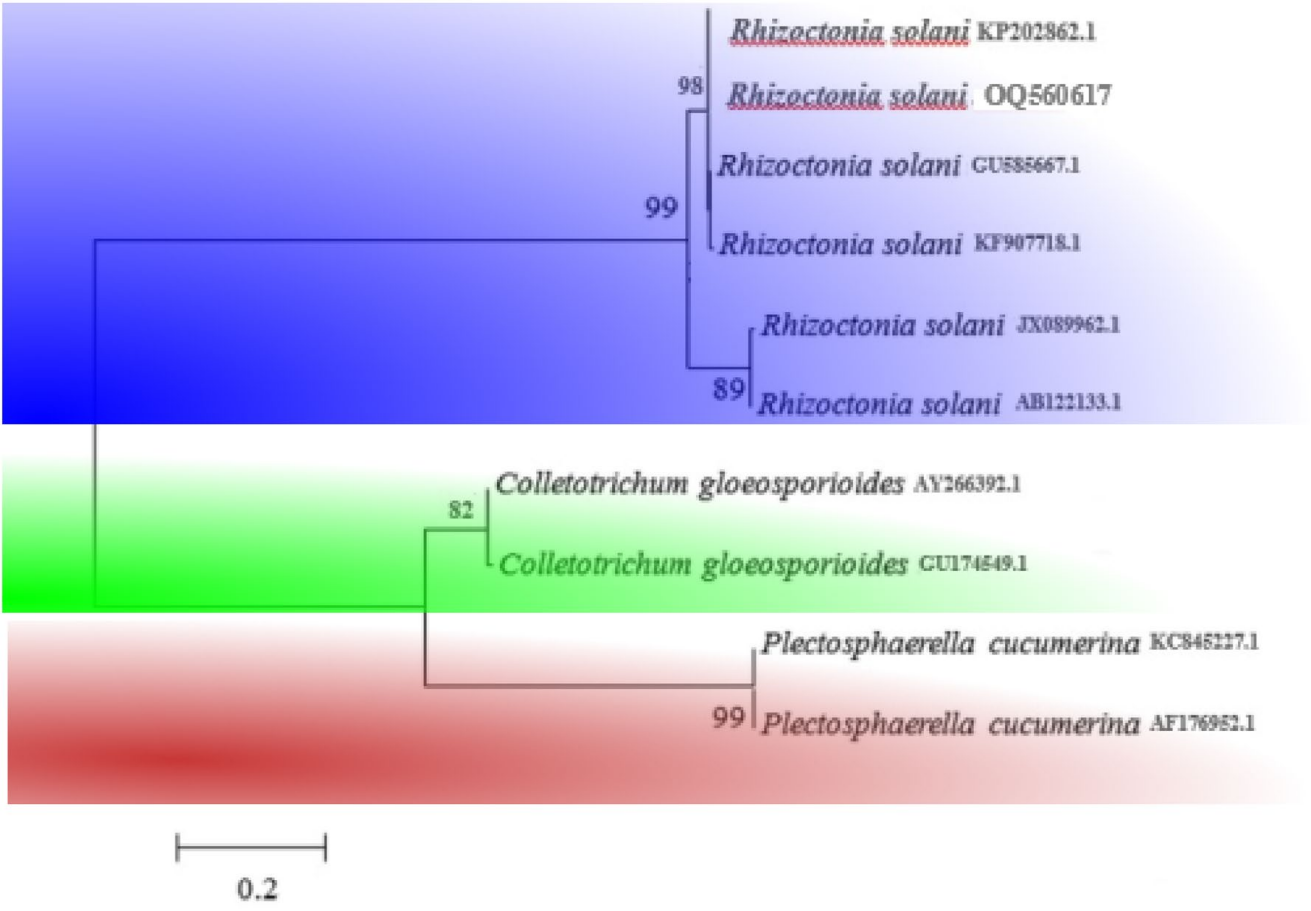
Identification of *R. solani* based on ITS phylogenetic tree. Maximum likelihood bootstrap values (MLB) higher than 50% (based on 1000 replicates) are displayed at the nodes.

In this work, the effects of silicon nanoparticles are determined on the target plant *Avena* *sativa* (variety NARC-Oats; certified seeds). The effects imposed by *R. solani* on this target plant help us to find out the changes of physio-chemical parameters in the alleviation of biotic stress. The ITS sequences of *R. solani* and a dataset of ITS based accessions were taken from published literature and downloaded from GenBank^55,56^. The sequences were aligned and edited using ClustalX 2.1^57^ and BioEdit^58^. The downloaded and newly generated sequences were aligned with MAFFT v. 10^59^ and manually edited at 593 positions. These sequences were used to construct the phylogenetic tree in MEGA 10.0 software using the maximum likelihood method with 1000 bootstrap replicates^37^. Branches with less than 50% bootstrap support collapsed in the obtained tree. The phylogenetic tree indicated the fungal species was *R. solani* and the consensus submitted to GenBank (Fig. 1) (accession No. OQ560617).

The in vivo experiment was conducted in a pot containing sandy loamy soil. The amount of sand, silt, and clay is also determined. The soil of pots was taken from the Botanical garden of LCWU. The quantity of sand, silt, and clay was 62%, 24%, and 14%, respectively. The soil texture used in this experiment was sandy loam. The EC and pH were 235 µscm^–1^**, **and 7.4 for sterilized soil. In vitro, experiments indicated that the germination rate was decreased in plants inoculated with fungus as compared to control plants and silicon-irrigated plants. Plants under certain conditions were capable to produce mineralized nano-materials naturally essential to their growth.

**Figure 2:**
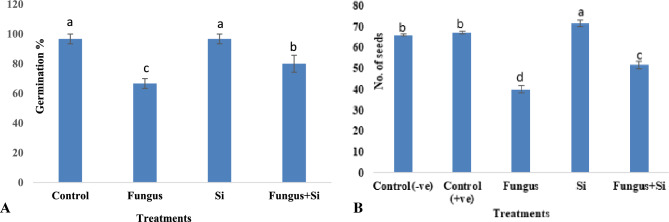
(**A**,**B**) Influence of silicon nano particles on *R.* *solani* stress on germination % of seedlings of *A. sativa* plant. Vertical bars showed standard error of means of replicates. Values having different letters showed significant variations analyzed through LSD testing at (P ≤ 0.05) differences.

**Figure 3:**
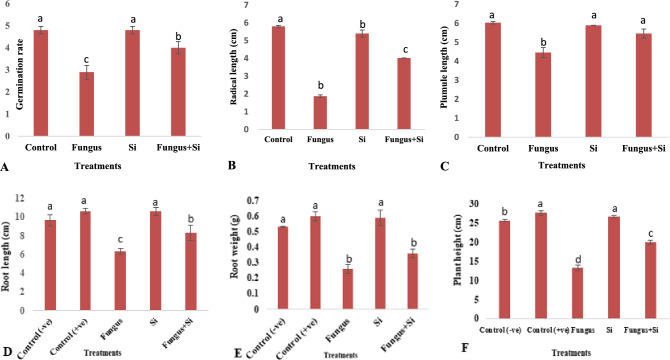
Influence of silicon nano particles on *R. solani* stress on germination rate (**A**); radical length (**B**); plumule length (**C**); root length (**D**); root weight (**E**) and plant height (**F**) of seedlings of *A. sativa* plant. Vertical bars showed standard error of means of replicates. Values having different letters showed significant variations analyzed through LSD testing at (P ≤ 0.05) differences.

As seed germination provides a suitable foundation for plant growth, development, and yield. Plants generally require silica to control biotic and abiotic stress^60^. In the current study, germination percentage and germination rate (66.6%) and (2.9 n/d) in the experimental set of fungus-affected soil, respectively as compared to the control, where germination percentage of 96.6% and germination rate was 4.8 n/d. The nanoparticles of this study significantly reduced the fungal effects. The soil amended with Si-nanoparticles enhanced the germination percentage (96.6%) and the germination rate was recorded (4.8 n/d) (Fig. 2A,B and 3A. In Fungus + Si experimental pots, Si particles revealed better results to minimize the stress of fungus i.e. germination percentage (80%) and germination rate was (4 n/d) (Fig. 2A,B and 3A). Generally, Si-unamended pots and *R. solani* inoculated plants are more vulnerable to fungal infection^61^. The negative effects were significantly minimized when plants were irrigated with silicon nanoparticles. Similar results regarding the positive effects of silicon to promote plant growth and control of *Rhizoctonia* and *Pythium* on soybeans were studied by^62^.

Results related to radical length and plumule length at the vegetative phase are shown in (Fig. 3B,C). Fungal stress caused adverse effects on plumule and radical length as compared to Fungus + Si inoculated plants. The silicon particles exhibited the best results in the alleviation of biotic stress caused by *R. solani* on both radical and plumule. The radical length and plumule length in the presence of Si particles was 5.4 cm and 5.89 cm, respectively. The severe effects of stress were observed in the treatment of *R. solani* alone i.e. radical length was 1.87 and plumule length was 4.46 as compared to Fungus + Si, where radical and plumule length was recorded at 4.02 and 5.46 respectively (Fig. 3B,C). In the current study, the Si nanoparticles played an important role in the reduction of biotic stress. Grau and Martinson^63^ reported that *R. solani* decreased the rate of hypocotyl elongation in some soybean varieties and subsequently delayed seedling growth. However, silicon is a positive article on the growth of plants. Earlier it was observed that the silicon treatment on plants increased the germination (index, rate, %), vitality index, plumule, and radical length of borage^64^.

Results showed that fungal stress significantly reduced the root length and weight as compared to the control (Fig. 3D,E). The root length and root weight in the presence of Si particles were 10.6 cm and 0.59 g, respectively. The drastic effects of stress were observed in the presence of *R. solani* i.e. root length was 6.3 cm and root weight was 0.26 g as compared to Fungus + Si where root length and root weight were recorded at 8.3 cm and 0.36 g, respectively.

The plant’s height was reduced when the soil was augmented with nano silica as compared to the control group. These particles facilitated the diseased plant to enhance the height as compared to plants that faced just biotic stress (Fig. 3F). In control it was 25.67 cm while fungal treatment reduced it to 13.3 cm and in Fungus + Si treatment it was increased upto 20 cm. Wang et al.^65^ reported the roles of Si in plant–microbe interactions. They improved the plants' potential and resistance by modifying the Si fertilizers application. This technique decreased the damage caused by pathogens and increased the yield of plants as well.

**Figure 4:**
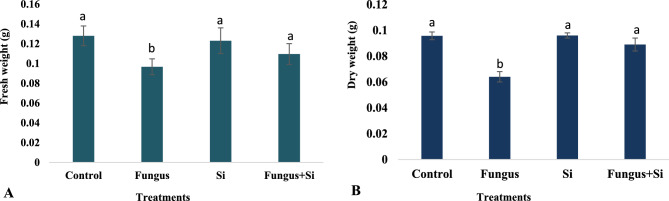
Influence of silicon nano particles on *R. solani* stress on Fresh weight (**A**) and Dry weight (**B**) of seedlings of *A. sativa* plant. Vertical bars showed standard error of means of replicates. Values having different letters showed significant variations analyzed through LSD testing at (P ≤ 0.05) differences.

A noteworthy decline in the fresh and dry weight of oats seedlings was observed in fungus-treated seeds, while the control group and silicon nanoparticles showed the highest rate (Fig. 4A,B). The effect of silicon nanoparticles on disease-treated seeds was slightly lower than the control group, which is a healthy sign. The dry weight of seedlings was recorded highest in silicon nanoparticles irrigated seeds i.e. 0.09 g, which showed that plants were readily affected by this micronutrient. Dry weight in fungus-treated seedlings had the least value (0.06 g), while seeds treated with Silicon + Fungus had 0.08 g dry weight (Fig. 4B). It showed that silicon increased the dry weight of seedlings. Similarly, silicon also improved fresh weight and dry weight of diseased plants (Fig. 4A). Liang et al.^62^ suggested that 200 mgL^–1^ concentration of Si in soybean inoculated with *Rhizoctonia* showed a significant increase in shoot biomass and root biomass compared to the plants inoculated only with *Rhizoctonia*. This is because Si taken up by the plant prevents pathogens from penetrating the root area as well as possibly inducing host defense reactions. Moreover, Azimi et al.^66^ also concluded that applying SiO_2_ nanoparticles increased the dry weight of shoot, root, and seedling of tall wheatgrass.

**Figure 5:**
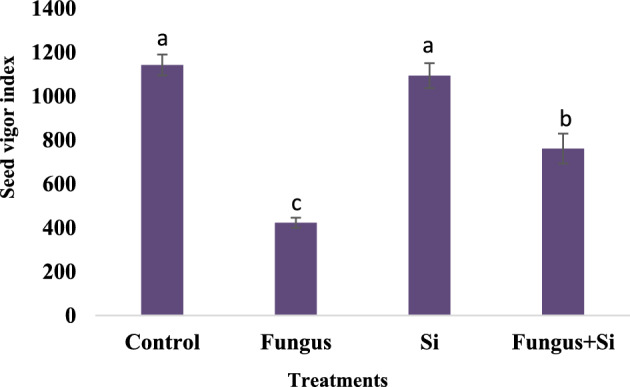
Influence of silicon nano particles on *R. solani* stress on seed vigour index of seedlings of *A. sativa* plant. Vertical bars showed standard error of means of replicates. Values having different letters showed significant variations analyzed through LSD testing at (P ≤ 0.05) differences.

Data regarding seed vigor index is shown in (Fig. 5). Results revealed that seed vigor decreased significantly under fungal stress in comparison to control and silicon nanoparticles. However, the silicon nanoparticles along with fungal application facilitated to overcome the stress by 35%. Siddiqui et al.^67^ studied the role of Si nanoparticles in the germination of tomato and reported that the application of 8 ppm of Si nanoparticles showed to be the greatest by giving the maximum values of seed germination and seedling vigor.

**Figure 6:**
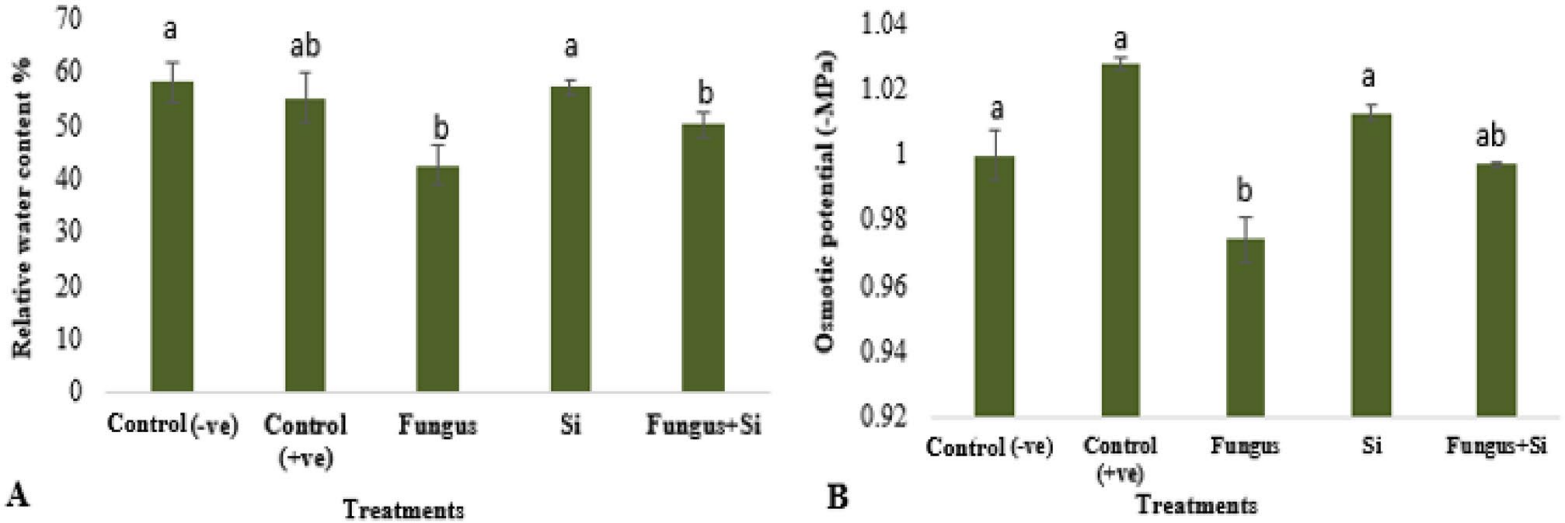
Influence of silicon nano particles on *R. solani* stress on relative water content (**A**) and osmotic potential (**B**) of *A. sativa* plant. Vertical bars showed standard error of means of replicates. Values having different letters showed significant variations analyzed through LSD testing at (P ≤ 0.05) differences.

Data for relative water contents in oats leaves at the reproductive stage in this study is presented in (Fig. 6A). At the reproductive stage, the relative water content was decreased by infection of *R. solani*. Relative water content was related to the fresh, dry, and turgid weight of plant leaves. The deficit of water in leaves indicated the severity of disease as relative water content decreased (42.5%) in Fungus treated plants and Fungus + Si (50.3%). Mali et al.^68^ reported that application of silicon (25–200 ppm) increased the relative yield, relative water contents, calcium, and potassium contents. Water content of leaves is affected under biotic stress as it works in photosynthesis and support in the transport of assimilates to other plant parts^69^.

The osmotic potential of leaves with silicon nanoparticles (1.01) was slightly less than the control group (1.02). These particles canceled out the negative effects caused by fungus by increasing the value close enough to itself (Fig. 6B). In fungus-treated plants the OP was 0.97, whereas 0.997 was observed in Fungus + Si experimental set. This study revealed that the osmotic potential of the leaves of oats was significantly reduced in fungal-inoculated plants. Ritchie et al.^70^ also observed that fungal stress declines the osmotic potential of leaves due to the accumulation of proline. Previous studies also explained the use of SiO_2_ nanoparticles increased the stability of cell membranes and photochemical efficiency, water holding capacity, and osmotic potential of the plants under water deficits, due to biotic stress^71^.

**Figure 7:**
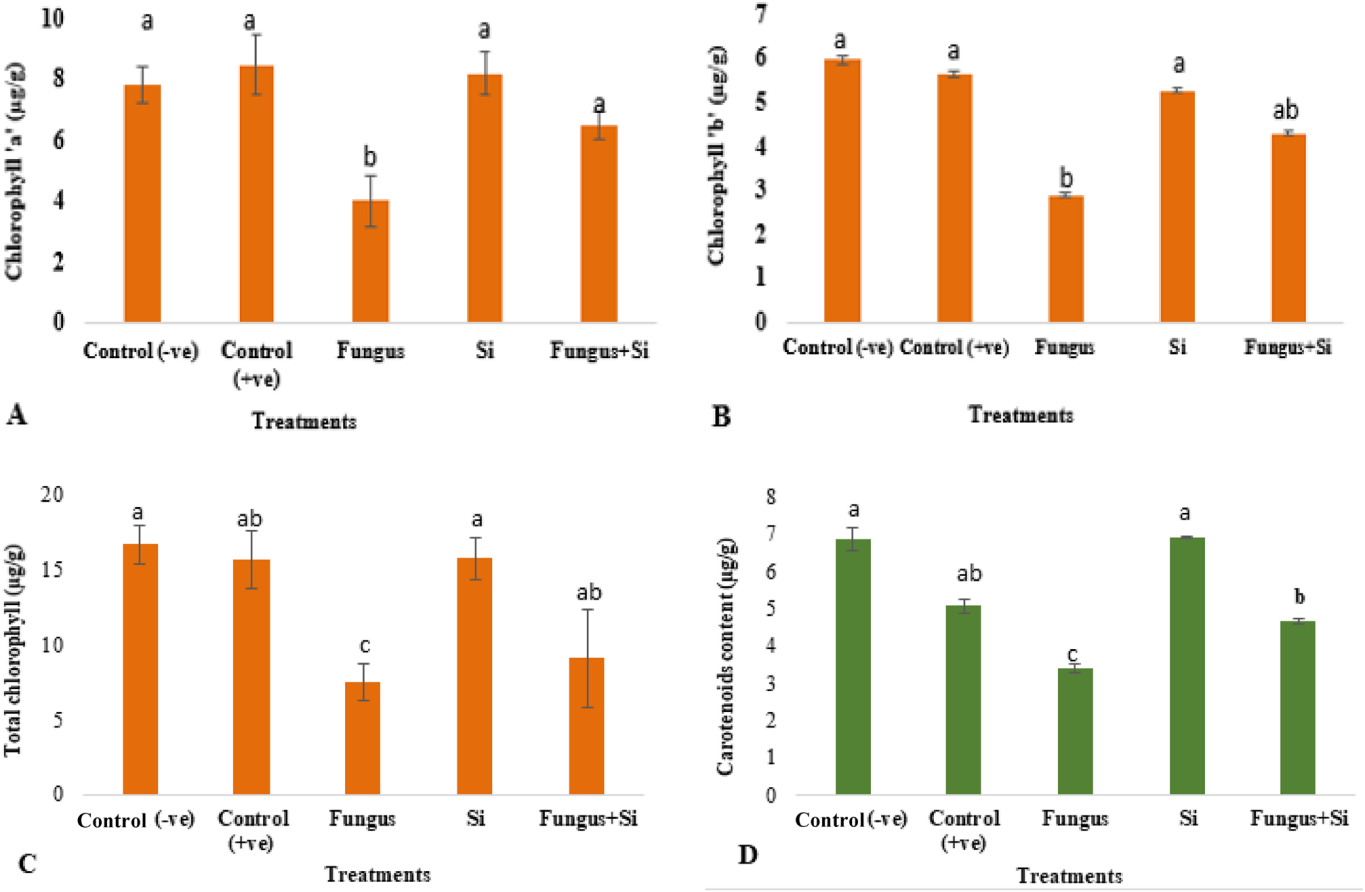
Influence of silicon nano particles on *R. solani* stress on chlorophyll ‘a’ (**A**); chlorophyll ‘b’(**B**); total chlorophyll (**C**) and carotenoid contents (**D**) of *A. sativa* plant. Vertical bars showed standard error of means of replicates. Values having different letters showed significant variations analyzed through LSD testing at (P ≤ 0.05) differences.

Chlorophyll a, b, total chlorophyll, and carotenoid contents decreased under fungal stress (Fig. 7A–D). Chlorophyll is an important pigment for photosynthesis particularly a and b^72^. Similarly, Barickman et al.^73^ also analyzed that infected tomato seedlings showed a decrease in chl. a chl. b, carotenoid contents as compared to control. Biotic stress leads to the loss of carotenoids and chlorophyll contents (a sign of leaf senescence)^74^. Maghsoudi et al.^75^ concluded similar results. According to them, the foliar application of silicon particles increased the membrane stability index (MSI), chlorophyll a, b (total chlorophyll), carotenoid contents, leaf relative water contents, and chlorophyll stability index in all wheat cultivars.

Chlorophyll a content was recorded highest in the positive control (8.49 µg/g). Silicon-treated plants and negative control exhibited slightly lower values of chlorophyll a i.e. 8.21 µg/g and 7.8 µg/g respectively than the positive control (Fig. 7A). A major decline was observed in chlorophyll b contents in fungus treated plant (2.9 µg/g) and a significant increase was found, when treated with nano silica solution (4.3 µg/g) (Fig. 7B). Data related to total chlorophyll content is also illustrated (Fig. 7C). Where positive control shows 15.65 µg/g and compare to treatments Fungus (7.47 µg/g) and Fungus + Si 9.09 µg/g. Results related to carotenoid content are represented in (Fig. 7D). Control shows higher carotenoid i.e. 5.07 µg/g as compared to Fungus (3.39 µg/g) and Fungus + Si (4.67 µg/g).

**Figure 8:**
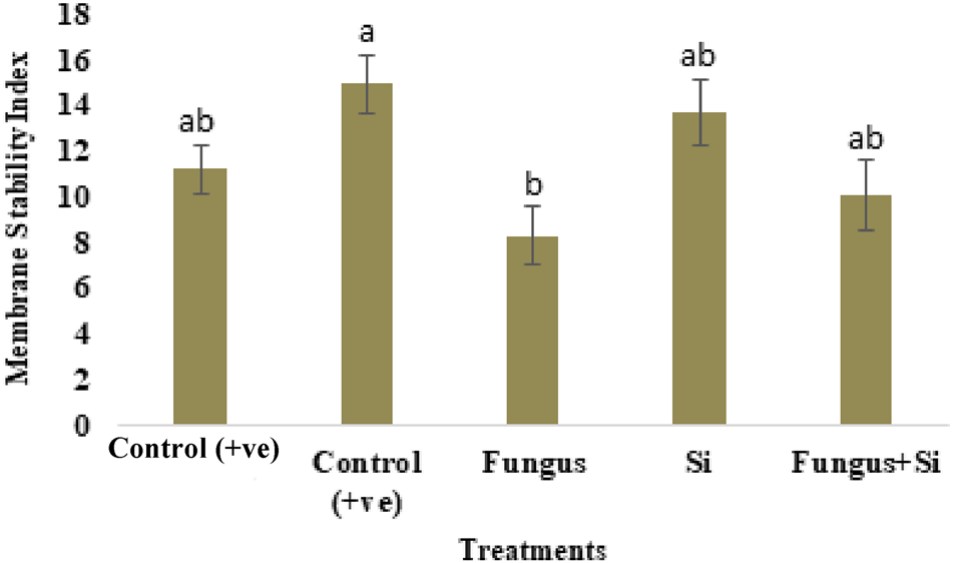
Influence of silicon nano particles on *R. solani* stress on membrane stability index of *A. sativa* plant. Vertical bars showed standard error of means of replicates. Values having different letters showed significant variations analyzed through LSD testing at (P ≤ 0.05) differences.

**Figure 9:**
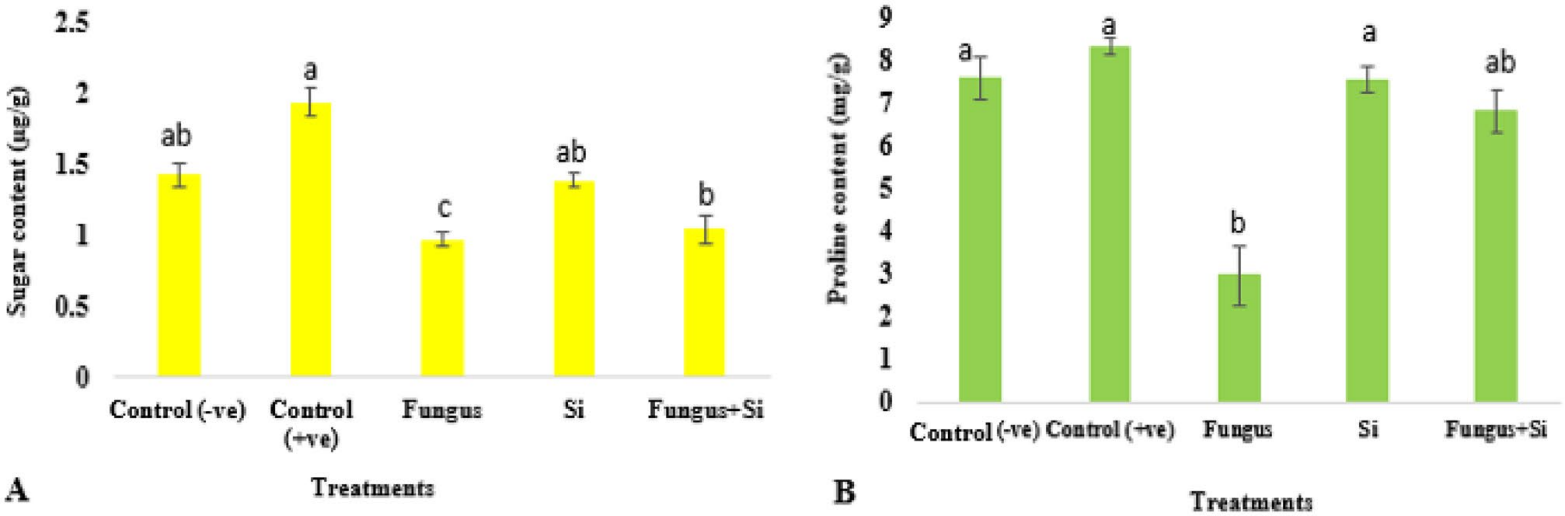
Influence of silicon nano particles on *R. solani* stress on sugar content (**A**) and proline content (**B**) of *A. sativa* plant. Vertical bars showed standard error of means of replicates. Values having different letters showed significant variations analyzed through LSD testing at (P ≤ 0.05) differences.

The membrane stability index (MSI) of oat leaves was higher in positive control as compared to negative control. The MSI was higher in the Fungus + Si treatment (10.07) as compared to Fungus (8.3) (Fig. 8). Sugar content reduced by fungal stress was overcome by silicon application. Control shows (1.94 µg/g**),** Fungus and Fungus + Si showed 0.97 and 1.05 µg/g of sugar (Fig. 9A). Dallagnol et al.^76^ presented the similar results. They revealed that a minimum Si concentration is required in leaf tissues of rice plants to evade the harmful effect of *Bipolaris oryzae* infection on sugar concentration and photosynthesis. The maximum level of Si concentration in leaves increased the soluble sugar concentration independently because soluble sugar and Si reduced the brown spot severity in rice plants.

The proline content of oat leaves was significantly increased under the biotic stress (Fig. 9B). In control plants' proline content was recorded as 7.6 mg/g, while fungus and fungus + Si showed 2.97 mg/g and 6.8 mg/g respectively. Jaleel et al.^77^ suggested that the addition of large quantities of proline (osmolytes) is an adaptive response in plants exposed to stressful environments. Silicon helps to alleviate the negative effect of stress by reducing the proline content.

**Figure 10:**
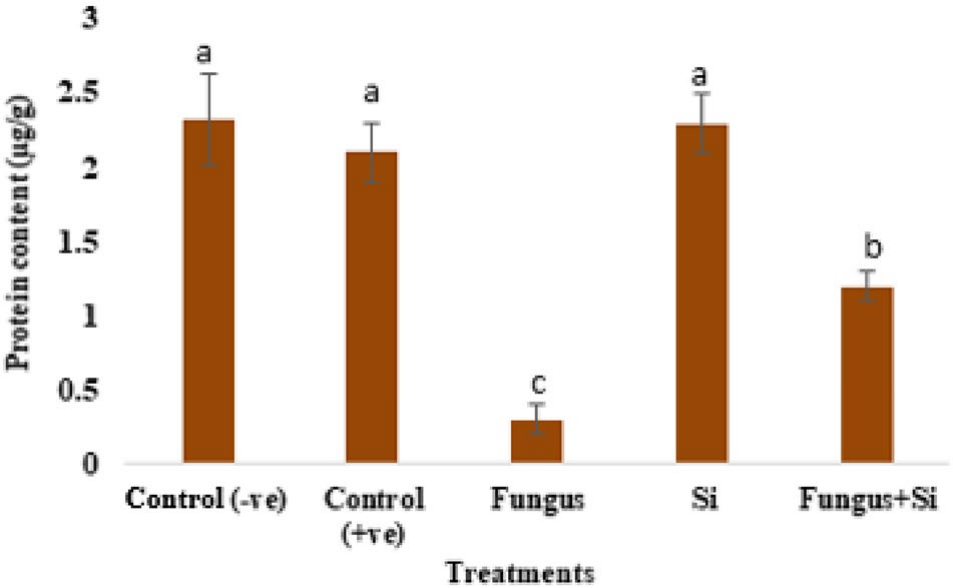
Influence of silicon nano particles on *R. solani* stress on protein content of *A. sativa* plant. Vertical bars showed standard error of means of replicates. Values having different letters showed significant variations analyzed through LSD testing at (P ≤ 0.05) differences.

Data regarding protein content in this work is presented in (Fig. 10). Control shows the highest Protein content as 2.32 µg/g and fungus with 0.3 µg/g as minimum protein content. Fungus + Si treatment showed increased protein content i.e. 1.2 µg/g as compared to fungus. Previous studies by Cai et al.^78^ suggested that Si-treated plants can considerably increase the antioxidant enzyme activities and production of antifungal compounds *e.g.,* proteins-related pathogenesis, phytoalexins, and products produced during phenolic metabolism.

**Figure 11:**
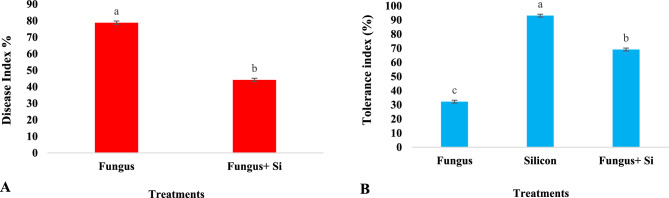
Influence of silicon nano particles on *R. solani* stress on disease index (**A**) and tolerance index (**B**) of *A. sativa* plant. Vertical bars showed standard error of means of replicates. Values having different letters showed significant variations analyzed through LSD testing at (P ≤ 0.05) differences.

In diseased index (DI), this study estimated the prevalence and amount of destruction caused by the infection. The DI was maximum (78.82%) when the fungus directly attacked the target plant. The results of DI and severity in diseased conditions on the target plant become reduced (44.2%), when the fungus was treated with Si nanoparticles. This particle saved the life of our plant. Root length and root weight were calculated to assess the disease index (Fig. 11). SNPs can considerably rise the resistance of crops to diverse kinds of ailments, including fungal (*Aspergillus niger*, *Alternaria solani*, *Colletotrichum* sp., and *Fusarium oxysporum*). Studies proved that SNPs increase resistance to *A. niger* and *F. oxysporum* in maize. SNPs induce *Arabidopsis* resistance to *P. syringae* by mediating the salicylic acid signaling pathway to defense resistance^79^.

The effects of fungal metabolites on the tolerance index of oat is also represented (Fig. 11B). Tolerance index of three seedlings was measured from a Petri dish after 7 days of growth and then root-shoot length was measured. The highest tolerance index (93.10%) was observed in silicon nanoparticle-treated plants in response to fungus, which had the lowest rate (32.24%). However, silicon nanoparticles enhanced the 30% tolerance index as compared to the Fungus + Si application. The results indicated that Si nanoparticles played a significant role to minimize the negativity caused by *R. solani*. The tolerance index of oat plants increased against the pathogenic effects of *R. solani*.

**Figure 12:**
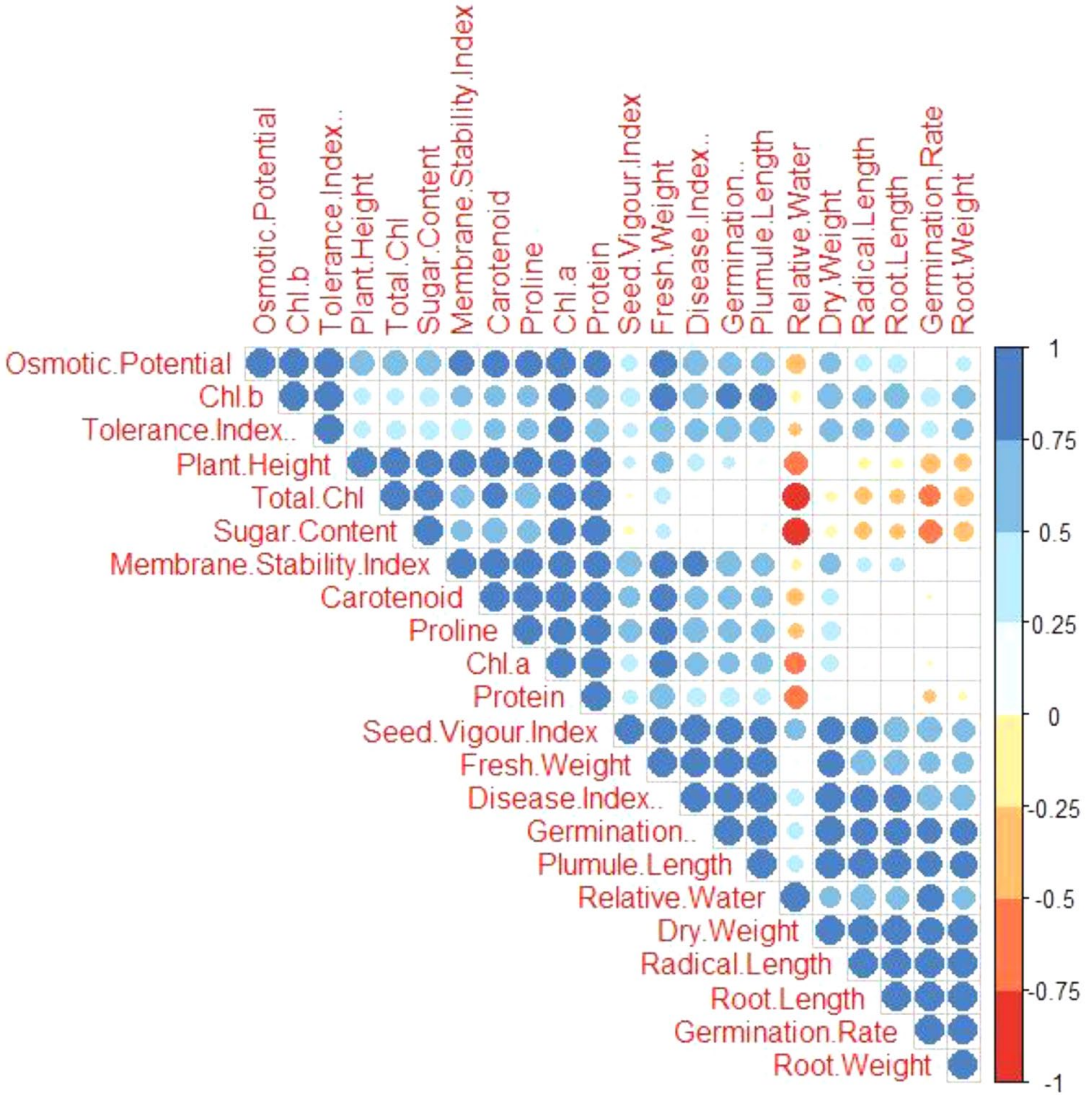
Correlation matrix of morphological, physiological, and biochemical parameters of *A. sativa* growing in *R. solani* contaminated soil following Si application.

After Si application, the Pearson correlation analysis was utilized to assess the relationship between the variables studied attributes of *A. sativa* raised in *R. solani* stress (Fig. 12). The osmotic potential in *A. sativa* shows a negative correlation with germination rate, and relative water while it is positively correlated with sugar, plant height, and tolerance index. Membrane stability in the plant is positively correlated with plumule length and disease index. While relative water has a negative correlation with the amount of total chlorophyll in plants (Fig. 12). The strong positive correlation was observed between proline content and sugar leaves under Si + Fungal treatment. The seed vigour index showed a negative correlation with all the morphological attributes.

**Figure 13:**
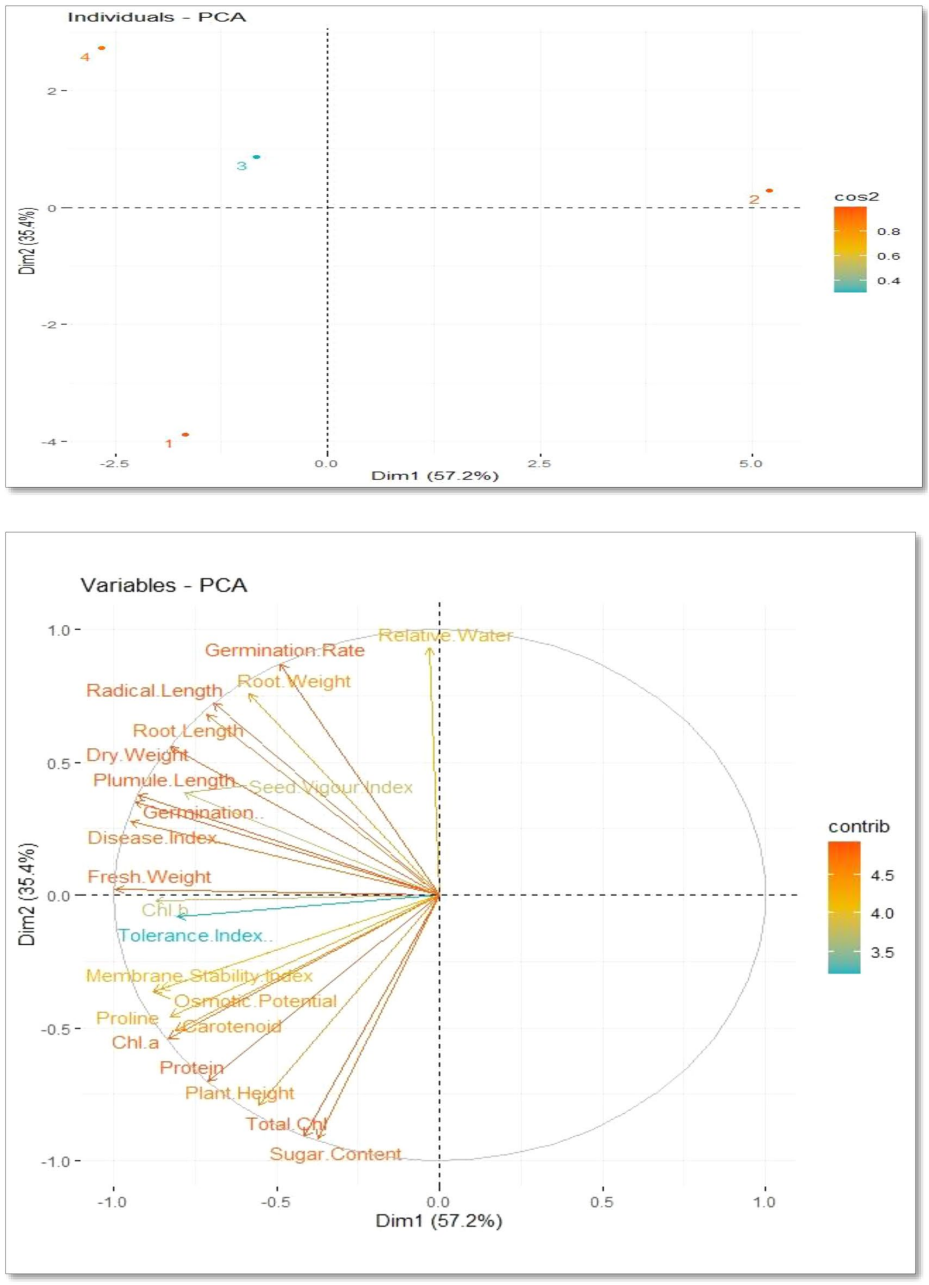
Principal component analysis loading plots showed a relationship between growth and physiological factors in *A. sativa* growing in fungal contaminated soil following Si application.

Following the application of fungus and Si, PCA loading plots were utilized to further highlight the association between the physiological characteristics and growth attributes of *A. sativa* growing in fungus (Fig. 13). With more than 88.8% of the whole database, the first two, Dim1 and Dim2 primary components the largest portion of all components are generated. Dim1 makes up 57.2% of the whole dataset, whereas Dim2 makes up 35.4%. The results showed that the relevant treatments were significantly distributed across the entire dataset. The distribution of all the dataset's variables demonstrated clearly that the effects of *R. solani* stress on various morpho-physio-biochemical characteristics were significant for all treatments examined in this study, while Si assisted in overcoming adversity.

The fungal content was most distant from all other regimens in the current investigation, demonstrating that it plays a substantial detrimental influence in *A. sativa* development and physiology. Germination rate, relative water, germination %, radical length plumule length, root length, root weight, fresh weight, dry weight, and seed vigour index are all positively correlated with PC2. Sugar content, total chlorophyll, plant height, protein, proline, membrane stability index, osmotic potential, Chl *a*, Chl *b*, carotenoid, protein content, and disease index %, shoot were all shown to be significantly inversely correlated (Fig. 13).

## Conclusion

This study concluded that Si nanoparticles significantly mitigate the adverse effects of *R. solani* in *A. sativa* as indicated by the various studied biochemical and physiological parameters. These nanoparticles can be used as silicon fertilizers, but further studies on their efficacy under field conditions and improvement in their synthesis are still needed. 

## Data Availability

Data available on personal request from the corresponding authors and the sequence of *R. solani* is deposited in GenBank-National Center for Biotechnology Information (NCBI), National Library of Medicine under accession No. OQ560617.
